# Supplementary material for: Taxol acts differently on different tubulin isotypes
Source: Commun Biol. 2023 Sep 16;6:946. doi: 10.1038/s42003-023-05306-y (PMC10505170; doi:10.1038/s42003-023-05306-y)
Supplement: Supplementary file 2 — Supplementary Information [file 42003_2023_5306_MOESM2_ESM.docx]

**Taxol acts differently on different tubulin isotypes**

Yean Ming Chew & Robert A. Cross

Centre for Mechanochemical Cell Biology, Warwick Medical School, Coventry CV4 7LA UK

Correspondence to: [r.a.cross@warwick.ac.uk](mailto:r.a.cross@warwick.ac.uk)

**Supplementary Figure 1** | **Sequences**. **a.** pBIG-α-β plasmid has Tn7 transposition sites that allow integration of tubulin genes into baculovirus genome. Plasmid carries sequences of codon-optimised tubulin genes (GeneArt) used for tubulin expression in *Spodoptera frugiperda*. **b.** Sequences. Sequences shown are coding region spanning L21 leader sequence and tag sequence, with a TAA stop codon. Highlighted in grey: L21 leader sequence; single underline: sequences encode for affinity tags (8x his for α tubulin and FLAG for β tubulin); double underline: linker (encodes for residue GGSGG).

**a**


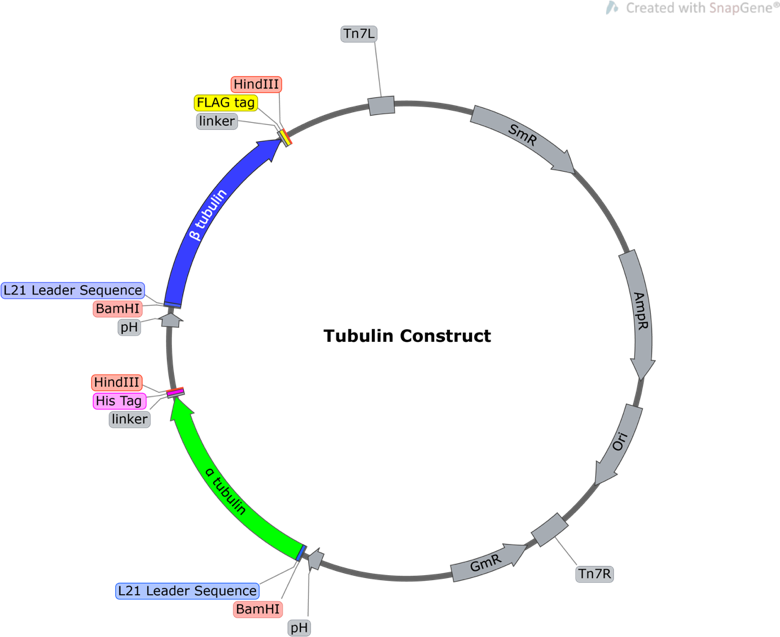


**b**

*Danio rerio* *tubb4b* encodes NP_942104

AACTCCTAAAAAACCGCCACCATGCGCGAGATCGTGCACTTGCAAGCTGGCCAGTGCGGCAACCAGATCGGTGCTAAGTTCTGGGAAGTGATCTCCGACGAGCACGGTATCGACCCCACCGGTTCTTACCACGGCGACTCTGACCTGCAGCTGGACCGTATCAACGTGTACTACAACGAGGCTACCGGTGGCAAATACGTGCCCCGTGCTGTGCTGGTGGACTTGGAGCCTGGTACTATGGACTCCGTGCGTTCCGGTCCTTTCGGCCAGATCTTCCGTCCTGACAACTTCGTGTTCGGCCAGTCCGGTGCTGGCAACAACTGGGCTAAGGGACACTACACCGAGGGTGCTGAACTGGTCGACTCTGTGTTGGACGTCGTGCGTAAAGAGGCTGAGTCCTGCGACTGCCTGCAGGGTTTCCAGCTGACTCACTCTCTCGGTGGTGGTACTGGTTCCGGCATGGGAACCCTGCTGATCTCCAAGATCCGTGAAGAGTACCCCGACCGCATCATGAACACCTTCTCCGTGGTGCCCTCACCTAAGGTGTCCGACACTGTGGTCGAGCCCTACAACGCTACCCTGTCTGTGCACCAGCTCGTCGAGAACACCGACGAGACTTACTGCATCGACAACGAAGCTCTGTACGACATCTGCTTCCGTACTCTGAAGCTGACCACTCCTACCTACGGCGACCTCAACCACCTGGTGTCTGCTACCATGTCCGGTGTCACCACTTGCCTGCGTTTCCCCGGTCAACTGAACGCTGACCTGAGAAAGCTGGCTGTGAACATGGTGCCCTTTCCAAGGCTGCACTTCTTTATGCCCGGTTTCGCTCCCCTGACCTCCAGGGGTTCTCAACAGTACAGGGCTCTGACCGTGCCTGAGCTGACCCAGCAAATGTTCGACGCTAAGAACATGATGGCTGCTTGCGACCCTCGTCACGGTCGTTACTTGACTGTGGCTGCTGTGTTCCGTGGTCGTATGTCCATGAAGGAAGTGGACGAGCAGATGCTGAACGTGCAGAACAAGAACTCCTCCTACTTCGTCGAGTGGATCCCCAACAACGTCAAGACCGCTGTGTGCGACATCCCTCCACGTGGCCTGAAAATGGCTGCTACCTTCATCGGCAACTCCACCGCTATCCAAGAGCTGTTCAAGCGCATCTCCGAGCAGTTCACCGCTATGTTCCGTCGCAAGGCTTTCCTGCACTGGTACACCGGCGAAGGCATGGACGAGATGGAATTCACCGAGGCCGAGTCCAACATGAACGACCTCGTGTCCGAGTACCAGCAGTACCAGGACGCTACCGCTGAGGAAGAGGGCGAGTTCGAAGAGGAAGGCGAAGAAGAACTGGCTGGCGGTTCTGGTGGCGACTACAAGGACGACGACGACAAATAA

*Danio rerio* *tuba1c* encodes NP_001098596

AACTCCTAAAAAACCGCCACCATGCGCGAGTGCATCTCCATCCATGTTGGCCAGGCTGGTGTCCAGATCGGCAACGCTTGTTGGGAGCTGTACTGCCTCGAGCACGGTATCCAGCCTGACGGACAGATGCCTTCCGACAAGACTATCGGTGGTGGCGACGACTCCTTCAACACCTTCTTCTCCGAAACCGGTGCTGGCAAGCACGTCCCAAGAGCTGTGTTCGTGGACCTCGAGCCTACCGTGATCGACGAAGTGCGTACCGGAACCTACCGTCAGCTGTTCCATCCTGAGCAGCTGATCACCGGCAAAGAGGACGCTGCTAACAACTACGCTCGTGGTCACTACACCATCGGCAAAGAAATCATCGACCTGGTGCTGGACCGTATCCGCAAGCTGGCTGACCAGTGTACTGGCCTGCAAGGTTTCCTGGTGTTCCACTCCTTCGGTGGCGGTACTGGTTCCGGTTTCACCTCTCTGCTGATGGAACGTCTGTCCGTGGACTACGGCAAGAAGTCCAAGCTCGAGTTCTCTATCTACCCCGCTCCTCAGGTGTCCACCGCTGTGGTGGAACCCTACAACTCTATCCTGACCACTCACACCACCTTGGAGCACTCCGACTGCGCTTTCATGGTGGACAACGAGGCTATCTACGACATCTGCCGTCGTAACCTGGACATCGAGCGTCCCACCTACACCAACCTGAACCGTCTGATCTCCCAGATCGTGTCCTCCATCACCGCTTCTCTGCGTTTCGACGGTGCTCTGAACGTGGACCTGACCGAGTTCCAGACCAACCTGGTGCCTTATCCTCGTATTCACTTCCCTCTGGCTACCTACGCTCCCGTGATCTCCGCTGAGAAGGCTTACCACGAGCAGCTGTCCGTCGCTGAGATCACCAACGCTTGCTTCGAGCCCGCTAACCAGATGGTCAAGTGCGACCCTCGTCACGGCAAGTACATGGCTTGCTGCCTGTTGTACCGTGGCGACGTGGTGCCTAAGGACGTGAACGCTGCTATCGCTACCATCAAGACCAAGCGTACCATCCAGTTCGTCGACTGGTGCCCTACCGGTTTCAAAGTGGGCATCAACTACCAGCCTCCAACCGTCGTTCCTGGTGGCGATCTGGCTAAGGTGCAACGTGCTGTGTGCATGCTGTCCAACACCACCGCTATCGCTGAGGCTTGGGCTCGTCTGGACCACAAGTTCGACCTGATGTACGCTAAGAGGGCTTTCGTGCATTGGTACGTCGGCGAAGGCATGGAAGAGGGCGAATTCTCTGAAGCTCGCGAGGACATGGCTGCTCTCGAGAAGGACTACGAGGAAGTGGGCGTCGACTCCATCGAAGGCGAGGGCGAAGAAGAGGGCGAAGAGTACGGTGGTTCTGGTGGTCACCATCACCACCACCATCATCACTAA

*Homo sapiens* *TUBB3* encodes NP_006077

AACTCCTAAAAAACCGCCACCATGCGCGAGATCGTGCACATCCAAGCTGGCCAGTGCGGCAACCAGATCGGTGCTAAGTTCTGGGAAGTGATCTCCGACGAGCACGGTATCGACCCCTCCGGAAACTACGTGGGAGACTCCGACCTGCAGCTGGAACGCATCTCCGTGTACTACAACGAGGCTTCCTCTCACAAATACGTGCCCCGTGCTATCCTGGTGGACCTGGAACCTGGTACTATGGACTCCGTGCGTTCCGGTGCTTTCGGTCACTTGTTCCGTCCTGACAACTTCATCTTCGGCCAGTCCGGTGCTGGCAACAACTGGGCTAAGGGACACTACACCGAGGGTGCTGAGCTGGTGGACTCTGTGCTGGACGTCGTGCGTAAAGAGTGCGAGAACTGCGACTGCCTGCAGGGTTTCCAGCTGACCCATTCTCTCGGTGGTGGTACTGGTTCCGGCATGGGAACCCTGCTGATCTCCAAAGTGCGCGAGGAATACCCCGACCGTATCATGAACACCTTCTCCGTGGTGCCCTCACCTAAGGTGTCCGACACTGTGGTCGAGCCCTACAACGCTACCCTGTCCATCCACCAACTGGTCGAGAACACCGACGAGACTTACTGCATCGACAACGAAGCTCTGTACGACATCTGCTTCCGTACTCTGAAGCTGGCTACCCCTACCTACGGCGACCTGAACCACCTGGTGTCTGCTACCATGTCCGGCGTGACCACCTCTCTGCGTTTCCCTGGTCAACTGAACGCTGACCTGCGCAAGCTGGCTGTGAACATGGTGCCCTTTCCAAGGCTGCACTTCTTTATGCCCGGTTTCGCTCCCCTGACCGCTCGTGGTTCTCAACAGTACAGGGCTCTGACCGTGCCTGAGCTGACTCAGCAGATGTTCGACGCTAAGAACATGATGGCTGCTTGCGACCCTCGTCACGGTCGTTACCTGACTGTGGCTACCGTGTTCCGTGGTCGTATGTCCATGAAGGAAGTGGACGAGCAGATGCTGGCTATCCAGTCCAAGAACTCCTCCTACTTCGTCGAGTGGATCCCCAACAACGTGAAGGTGGCCGTGTGCGACATTCCTCCACGTGGCCTGAAGATGTCCTCCACCTTCATCGGCAACTCCACCGCTATCCAAGAGCTGTTCAAGAGGATCTCCGAGCAGTTCACCGCTATGTTCCGTCGCAAGGCTTTCCTGCACTGGTACACCGGCGAAGGCATGGACGAGATGGAATTCACCGAGGCTGAGTCCAACATGAACGACCTCGTGTCCGAGTACCAGCAGTACCAGGACGCTACCGCTGAAGAGGAAGGCGAGATGTACGAGGACGACGAGGAAGAGTCTGAGGCTCAGGGTCCTAAAGGTGGTAGCGGTGGCGACTACAAGGACGATGACGACAAATAA

*Homo sapiens* *TUBA1B* encodes NP_006073

AACTCCTAAAAAACCGCCACCATGCGCGAGTGCATCTCCATCCATGTTGGCCAGGCTGGTGTCCAGATCGGCAACGCTTGTTGGGAGCTGTACTGCCTCGAGCACGGTATCCAGCCTGACGGACAGATGCCTTCCGACAAGACTATCGGTGGTGGCGACGACTCCTTCAACACCTTCTTCTCCGAAACCGGTGCTGGCAAGCACGTCCCAAGAGCTGTGTTCGTGGACCTCGAGCCTACCGTGATCGACGAAGTGCGTACCGGAACCTACCGTCAGCTGTTCCATCCTGAGCAGCTGATCACCGGCAAAGAGGACGCTGCTAACAACTACGCTCGTGGTCACTACACCATCGGCAAAGAAATCATCGACCTGGTGCTGGACCGTATCCGCAAGCTGGCTGACCAGTGTACTGGCCTGCAAGGTTTCCTGGTGTTCCACTCCTTCGGTGGCGGTACTGGTTCCGGTTTCACCTCTCTGCTGATGGAACGTCTGTCCGTGGACTACGGCAAGAAGTCCAAGCTCGAGTTCTCTATCTACCCCGCTCCTCAGGTGTCCACCGCTGTGGTGGAACCCTACAACTCTATCCTGACCACTCACACCACCTTGGAGCACTCCGACTGCGCTTTCATGGTGGACAACGAGGCTATCTACGACATCTGCCGTCGTAACCTGGACATCGAGCGTCCCACCTACACCAACCTGAACCGTCTGATCTCCCAGATCGTGTCCTCCATCACCGCTTCTCTGCGTTTCGACGGTGCTCTGAACGTGGACCTGACCGAGTTCCAGACCAACCTGGTGCCTTATCCTCGTATTCACTTCCCTCTGGCTACCTACGCTCCCGTGATCTCCGCTGAGAAGGCTTACCACGAGCAGCTGTCCGTCGCTGAGATCACCAACGCTTGCTTCGAGCCCGCTAACCAGATGGTCAAGTGCGACCCTCGTCACGGCAAGTACATGGCTTGCTGCCTGTTGTACCGTGGCGACGTGGTGCCTAAGGACGTGAACGCTGCTATCGCTACCATCAAGACCAAGCGTTCCATCCAGTTCGTCGACTGGTGCCCTACCGGTTTCAAAGTGGGCATCAACTACCAGCCTCCAACCGTCGTTCCTGGTGGCGATCTGGCTAAGGTGCAACGTGCTGTGTGCATGCTGTCCAACACCACCGCTATCGCTGAGGCTTGGGCTCGTCTGGACCACAAGTTCGACCTGATGTACGCTAAGAGGGCTTTCGTGCATTGGTACGTCGGCGAAGGCATGGAAGAGGGCGAATTCTCTGAAGCTCGCGAGGACATGGCTGCTCTCGAGAAGGACTACGAGGAAGTGGGCGTCGACTCCGTTGAAGGCGAGGGCGAAGAAGAGGGCGAAGAGTACGGTGGTTCTGGTGGTCACCATCACCACCACCATCATCACTAA

*Homo sapiens* *TUBB4B* encodes NP_006079

AACTCCTAAAAAACCGCCACCATGCGCGAGATCGTGCACTTGCAAGCTGGCCAGTGCGGCAACCAGATCGGTGCTAAGTTCTGGGAAGTGATCTCCGACGAGCACGGTATCGACCCTACCGGTACTTACCACGGCGACTCCGACCTGCAGCTGGAACGTATCAACGTGTACTACAACGAGGCTACCGGTGGCAAATACGTGCCCCGTGCTGTGCTGGTGGACTTGGAGCCTGGTACTATGGACTCCGTGCGTTCCGGTCCTTTCGGCCAGATCTTCCGTCCTGACAACTTCGTGTTCGGCCAGTCCGGTGCTGGCAACAACTGGGCTAAGGGACACTACACCGAGGGTGCTGAACTGGTCGACTCTGTGTTGGACGTCGTGCGTAAAGAGGCTGAGTCCTGCGACTGCCTGCAGGGTTTCCAGCTGACTCACTCTCTCGGTGGTGGTACTGGTTCCGGCATGGGAACCCTGCTGATCTCCAAGATCCGTGAAGAGTACCCCGACCGTATCATGAACACCTTCTCCGTGGTGCCCTCACCTAAGGTGTCCGACACTGTGGTCGAGCCCTACAACGCTACCCTGTCTGTGCACCAGCTCGTCGAGAACACCGACGAGACTTACTGCATCGACAACGAAGCTCTGTACGACATCTGCTTCCGTACTCTGAAGCTGACCACTCCTACCTACGGCGACCTGAACCACCTGGTGTCTGCTACCATGTCCGGTGTCACCACTTGCCTGCGTTTCCCCGGTCAACTGAACGCTGACCTGAGAAAGCTGGCTGTGAACATGGTGCCCTTTCCAAGGCTGCACTTCTTTATGCCCGGTTTCGCTCCCCTGACCTCCAGGGGTTCTCAACAGTACAGGGCTCTGACCGTGCCTGAGCTGACCCAGCAAATGTTCGACGCTAAGAACATGATGGCTGCTTGCGACCCTCGTCACGGTCGTTACTTGACTGTGGCTGCTGTGTTCCGTGGTCGTATGTCCATGAAGGAAGTGGACGAGCAGATGCTGAACGTGCAGAACAAGAACTCCTCCTACTTCGTCGAGTGGATCCCCAACAACGTCAAGACCGCTGTGTGCGACATCCCTCCACGTGGCCTGAAGATGTCCGCTACCTTCATCGGCAACTCCACCGCTATCCAAGAGCTGTTCAAGCGCATCTCCGAGCAGTTCACCGCTATGTTCCGTCGCAAGGCTTTCCTGCACTGGTACACCGGCGAAGGCATGGACGAGATGGAATTCACCGAGGCCGAGTCCAACATGAACGACCTCGTGTCCGAGTACCAGCAGTACCAGGACGCTACCGCTGAGGAAGAGGGCGAGTTCGAAGAAGAGGCCGAAGAGGAAGTGGCTGGCGGTTCTGGTGGCGACTACAAGGACGACGACGACAAATAA

| **a** | **b** |
| --- | --- |
| 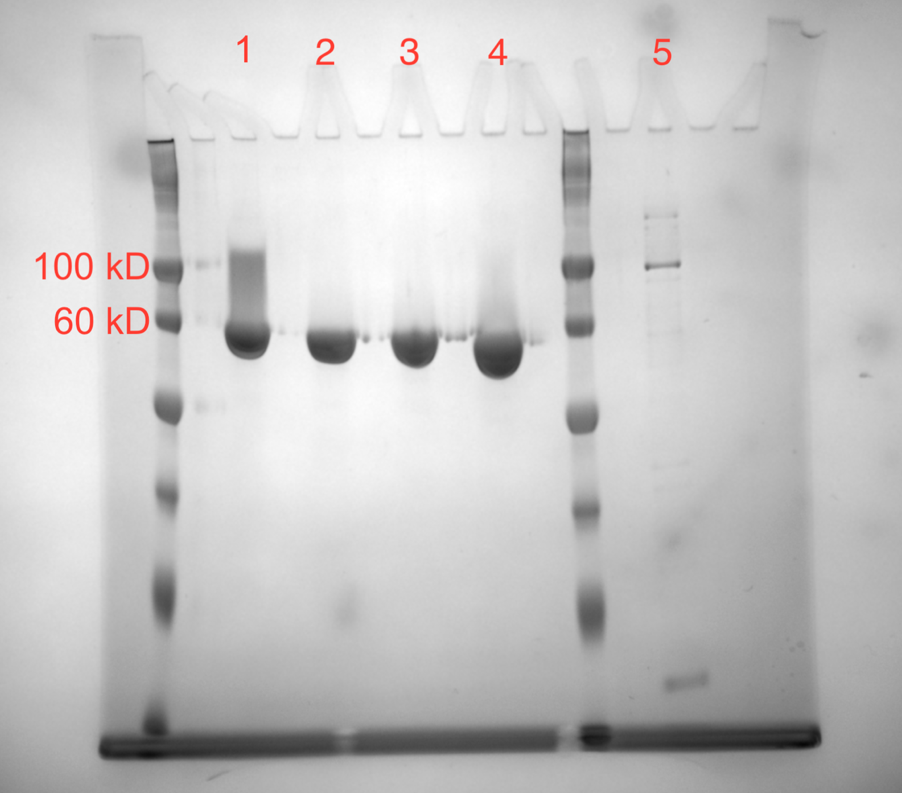 | **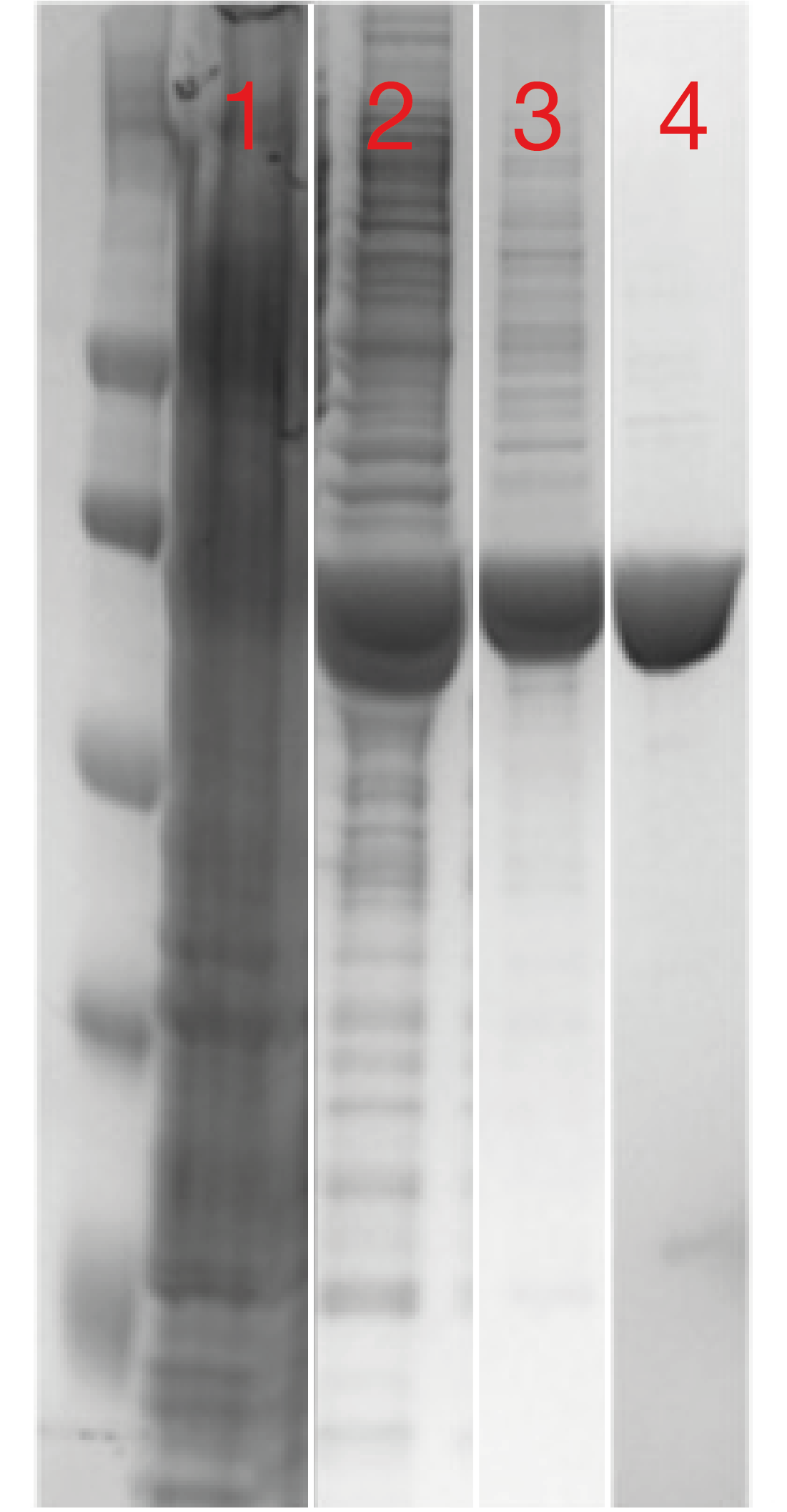** |

**Supplementary Figure 2 | Purity of single isotype tubulins.** Coomassie stained SDS gel of single isotype tubulins purified by double tag affinity chromatography. **a,** Purified single isotype tubulins 1: *Homo sapiens* α1β3; 2: *Homo sapiens* α1β4 tubulin; 3: *Danio rerio* α1β4 tubulin; 4: porcine brain tubulin; 5: *Drosophila melanogaster* full length kinesin-1. **b**, Representative purity through the steps of the purification process, here of *Homo sapiens* α1β3 tubulin (same markers). 1: cell homogenate; 2: Ni column eluate 3: FLAG column eluate, 4: concentrated peak fractions from ion exchange step. Same markers.


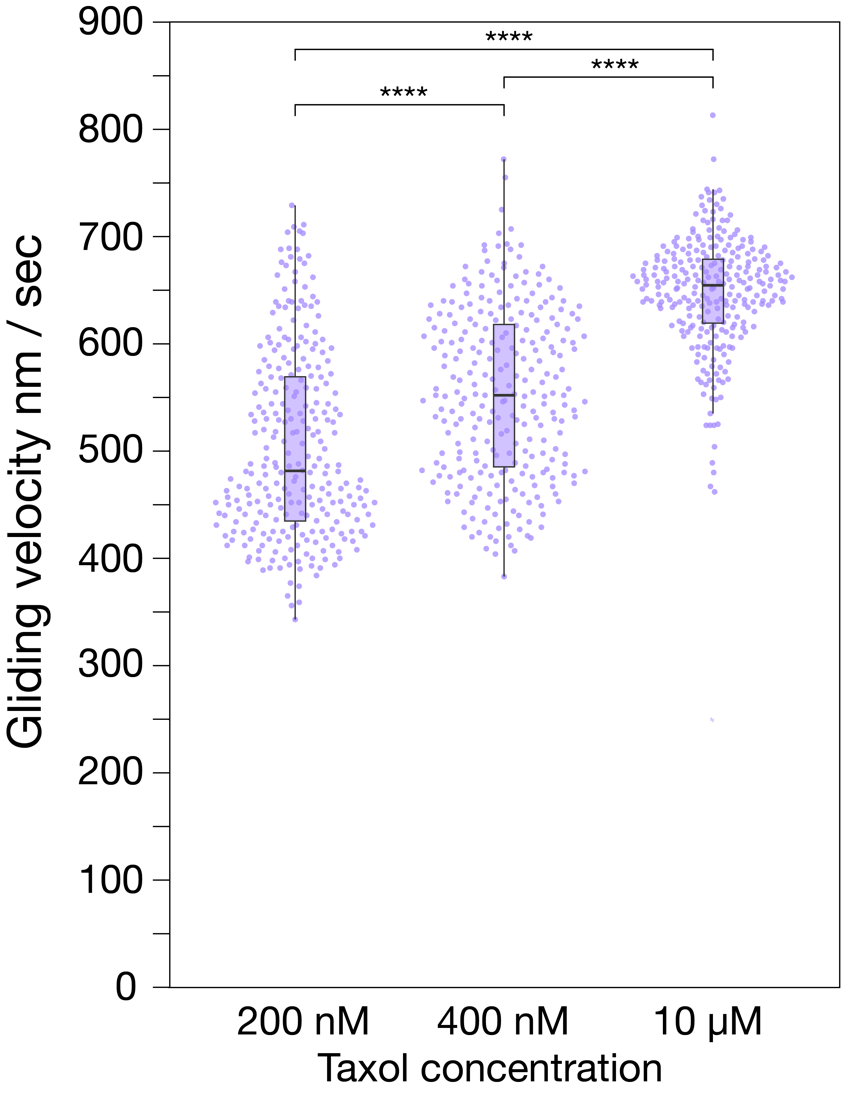


**Supplementary Figure 3** | **Gliding velocity of porcine brain microtubule over kinesin-coated surface in various taxol concentrations**. Increasing taxol concentration accelerates microtubule gliding. Boxes indicate interquartile range, whiskers mark SD, horizontal bar marks population median. All the data are plotted. Significance is * p < 0.05, ** p < 0.01, *** p < 0.001 and ****p < 0.0001.
