# Supplementary material for: Taxol acts differently on different tubulin isotypes
Source: Commun Biol. 2023 Sep 16;6:946. doi: 10.1038/s42003-023-05306-y (PMC10505170; doi:10.1038/s42003-023-05306-y)
Supplement: Supplementary file 3 — Description of Additional Supplementary Files [file 42003_2023_5306_MOESM3_ESM.docx]

**Description of Additional Supplementary Files**

**File name:** Supplementary Data 1

**Description:** The source data behind the graphs in the paper

**File name:** Supplementary Movie 1

**Description:** Hs α1β3 microtubules do not show prominent kinks when assembled in 500 nM taxol.

**File name:** Supplementary Movie 2

**Description:** Kinks develop in Dr α1β4 microtubules assembled in 500 nM taxol.

**File name:** Supplementary Movie 3

**Description:** taxol-induced kinking is reversible. Kinks develop in Dr α1β4 microtubules assembled in 500 nM taxol. Kinks relax upon washing out taxol using free tubulin solution without taxol.

**File name:** Supplementary Movie 4

**Description:** Segmented isotype microtubules gliding over a Drosophila full length kinesin-1 coated surface. With slower-gliding Hs α1β3 segments leading, faster gliding trailing segments, assembled from Dr α1β4 tubulin, squiggle up in 2 mM ATP (left). The squiggles become more prominent on addition of equimolar ADP (right).

**File name:** Supplementary Movie 5

**Description:** Segmented isotype microtubules gliding over a Drosophila full length kinesin-1 coated surface. With slower-gliding Hs α1β3 segments leading, faster gliding trailing segments, assembled from porcine brain tubulins, squiggle up in 2 mM ATP (left). The squiggles become more prominent on addition of equimolar ADP (right).

**File name:** Supplementary Movie 6

**Description:** Sinuosity of mosaic microtubules sliding over a *Drosophila* full-length kinesin coated surface. Microtubules prepared from a mixture of Hs α1β3 and Dr α1β4 tubulins (**b**) appear more sinuous than those built from only single isotype α1β3 (**a**) or α1β4 (**c**) tubulins. Stock solutions of both isotypes were supplemented with 5% fluorescent porcine brain tubulin to allow visualisation.  

**File name:** Supplementary Movie 7

**Description:** Molecular graphics showing the positions of residues that differ between β3 and β4 isotypes. Differences at surface-exposed residue positions (orange) cluster at the extreme C-terminus (not shown) and in the H1-S2 and H2-S3 loops, which engage the neighbouring M-loop (pink, TARGSQQYRA). Similar links form between α tubulins (not highlighted). α tubulin in cyan, β tubulin in purple. Taxol occupies a binding pocket (here unoccupied) in β tubulin directly abutting the M-loop. Graphic made in Chimera X41, using 6EVW.pdb.

M-loop (pink, TARGSQQYRA). Similar links form between α tubulins (not highlighted). a 35 tubulin in cyan, b tubulin in purple. c, taxol occupies a binding pocket in β tubulin so as to 36 directly abut the M-loop. Graphic made in Chimera X41, using 6EVW.pdb.

37

38
